# Acute exudative paraneoplastic polymorphous vitelliform maculopathy as a first presentation of metastatic melanoma

**DOI:** 10.1016/j.jdcr.2026.08.021

**Published:** 2026-08-23

**Authors:** James Ralph, Alan Hopkins, Gregg Murray, Claudine Howard-James, Claire Quigley, Helena Yoo, Michael Maguire, Stephen Crowther, Fergal Kelleher, Caroline Bailly, Asad Salim

**Affiliations:** a Department of Dermatology, Tallaght University Hospital, Dublin, Ireland; b Department of Medical Ophthalmology, Royal Victoria Eye and Ear Hospital, Dublin, Ireland; c Department of Histopathology, Tallaght University Hospital, Dublin, Ireland; d Department of Medical Oncology, Tallaght University Hospital, Dublin, Ireland

**Keywords:** checkpoint inhibitor, immunotherapy, maculopathy, melanoma, metastatic, paraneoplastic

## Case report

### Case 1

An 80-year-old man, with a history of melanomas, presented to ophthalmology and reported new-onset blurred vision in the last month.

Ophthalmic history included stable glaucoma. Fundoscopy demonstrated multiple bilateral serous areas of retinal detachment with yellowish vitelliform lesions scattered across both maculae (Fig 1, *A*). Fundus autofluorescence, used to determine pathologic changes of the retina and retinal pigmented epithelium, showed multiple bilateral areas of dark gray hypofluorescence foci around the maculae (Fig 1, *B*). Optical coherence tomography showed gross edema below the maculae bilaterally with normal choroidal thickness (Fig 1, *C*).

**Fig 1:**
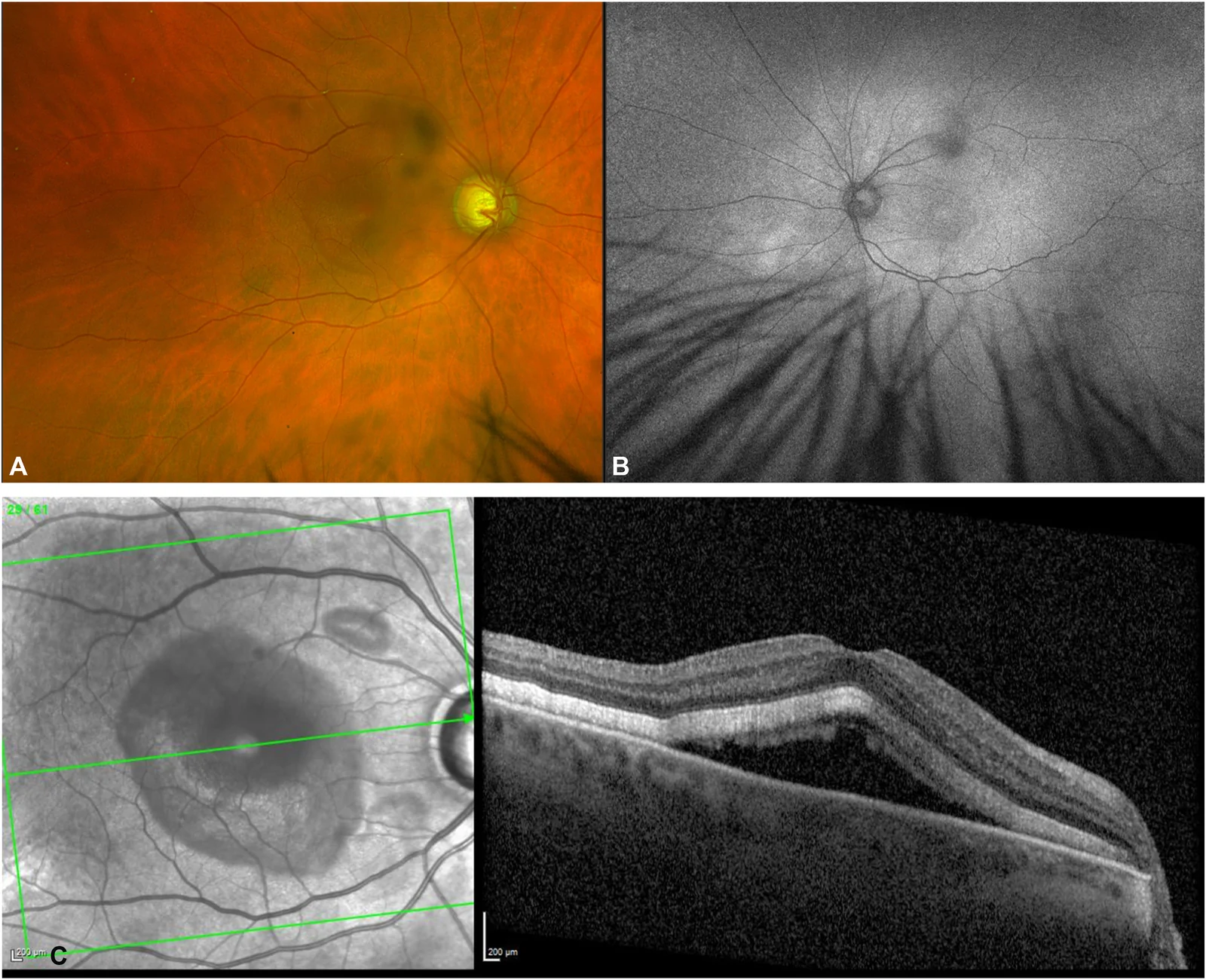
**A,** Fundoscopy showing multiple *yellow* areas with a honeycomb appearance. **B,** Fundus autofluorescence shows several *gray* (hypofluorescence) areas surrounding the maculae indicating retinal pigment epithelium damage. **C,** Optical coherence tomography shows accumulation of fluid with development of a hypopyon-like area. Cross-section shows gross edema below the fovea. (right eye).

The symptoms and investigative findings were consistent with acute exudative polymorphous vitelliform maculopathy. This is a rare immune-mediated maculopathy that can be idiopathic or paraneoplastic (acute exudative polymorphous vitelliform maculopathy). No choroidal melanoma was detected by ophthalmology. He was referred to dermatology for a skin check.

Relevant medical history included 3 thin melanomas, benign prostate hyperplasia, and a transient ischemic attack.

He previously attended dermatology for melanoma in situ (MIS) on his left scapula in 2013 and a clinical stage IA, Breslow thickness 0.45 mm, from his left cheek in 2015. He was subsequently discharged after clear surgical margins and appropriate follow-up. He was diagnosed with a third melanoma (MIS) on his mid back in 2023 (Fig 2). This was also managed surgically with clear margins. His brother also had a melanoma. The patient was referred to the cancer genetics service.

**Fig 2:**
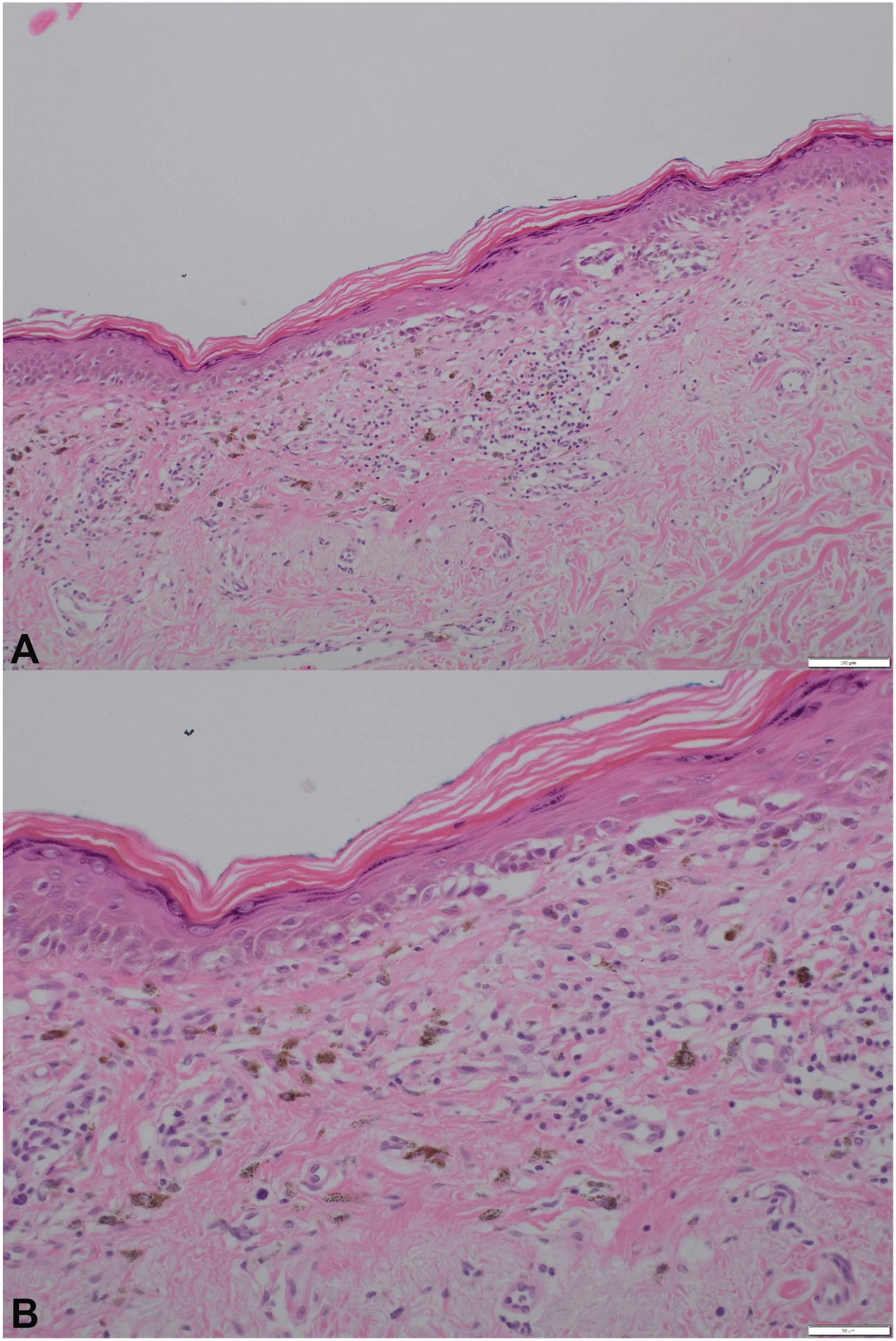
Melanoma in situ, middle portion of the left side of the back, September 2023. Dermis shows regression and probable residual invasive melanoma (single cells)—0.2 mm. (Original magnifications: **A, ×**10; **B, ×**20).

He had no clinically concerning skin lesions on examination. A urology examination and prostate-specific antigen was normal. Computed tomography of the thorax, abdomen, and pelvis showed diffuse metastasis (of unknown primary) with lesions in the liver, retroperitoneum, and abdomen. He had a magnetic resonance imaging of the brain several weeks beforehand, for a transient ischemic attack, which showed no suspicious lesions. There had been no weight loss and he was otherwise well. Systemic blood tests, including lactate dehydrogenase, were normal.

Core biopsy of a presumed hepatic metastasis confirmed metastatic melanoma on histopathology (Fig 3). Molecular testing demonstrated no mutations in BRAF, c-kit, N-RAS, or NTRK1/2/3 gene fusions. He commenced nivolumab on a 28-day schedule. One month later, brain metastases were diagnosed and stereotactic radiotherapy was administered. He developed hypophysitis and myocarditis as immune-related adverse events but was able to continue treatment. A repeat computed tomography of the thorax, abdomen, and pelvis and magnetic resonance imaging brain at 6 months demonstrated a partial response. At 8 months postdiagnosis, he is feeling well and is physically independent. His eyesight is stable.

**Fig 3:**
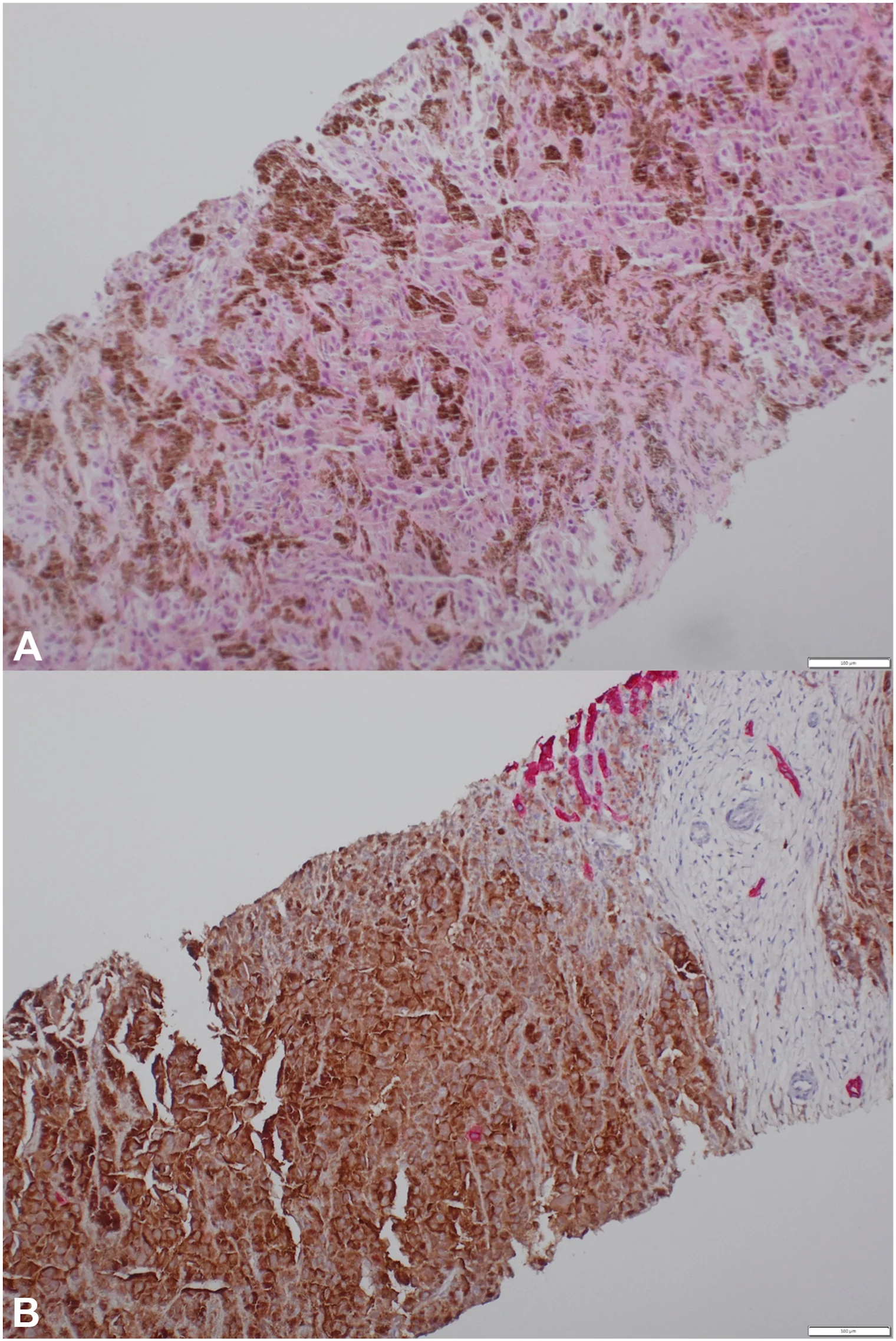
**A,** Liver core biopsy, original magnification: **×**10. Liver completely replaced by metastatic melanoma. **B,** Liver core biopsy using an immunohistochemical double stain; S100 stains melanoma *brown*, whereas AE1/3 stains residual hepatocytes *red*. (**A,** Original magnification: **A,** ×10.) (**B,** S100 and AE1/3 double stain; original magnification: **B, ×**10).

## Discussion

Acute exudative polymorphous vitelliform maculopathy is a rare condition affecting the macula. Diagnosis is made by clinical history and ophthalmology examination. Patients experience acute decreased visual acuity, visual distortion (blurriness), photopsia, nyctalopia, and/or metamorphopsia and headache. It is characterized by imaging findings demonstrating retinal pathology on fundoscopy, fundus autofluorescence, and optical coherence tomography.^1^ (Fig 1 *A* to *C*).

It is thought to be an immune-mediated condition due to an attack on the retinal pigmented epithelium. It is broadly classified as idiopathic or paraneoplastic. The idiopathic version has no clear association but has been reported with trauma, a variety of infections (predominantly viral) or idiosyncratic. It tends to self-resolve over a few months. The paraneoplastic form is most associated with cutaneous or choroidal melanoma. Carcinomas of the breast, lung, and colon have been reported. An even rarer third form, drug-induced, may be possible as some cases have been reported after the initiation of immunotherapy.^1^^,^^2^

Retinopathies and maculopathies are known to have a paraneoplastic association. The proposed mechanisms are molecular mimicry and cross-reaction between neoplastic antigens and various retinal proteins. Cross-reactivity between choroidal self-melanocytes and malignant melanoma cells may be why it occurs more often in the setting of melanoma.^3^

Treatment for acute exudative polymorphous vitelliform maculopathy is more straightforward than for other retinopathies. Prioritizing tumor control and reducing tumor burden improves the condition. The immune-mediated damage is known to be reversible with a good prognosis. Due to the rarity, there is no clear consensus on treatment although steroids are most commonly used.^1^^,^^2^

Identifying a proliferative mutation in a melanoma allows for the use of targeted therapy in addition to immunotherapy. This patient’s metastatic deposit, and most recent historical MIS from 2023, showed no detectable mutations to be exploited. Single agent nivolumab was selected as the lowest-risk option to minimize possible drug-induced side effects on top of the already established paraneoplastic effect. Combination therapy was avoided because ocular adverse events to immune checkpoint inhibitors have been suggested with mouse models showing retinopathy changes to ipilimumab.^4^

There is no clear data on the risk of in situ disease later becoming metastatic, with incidences being limited to case reports. Signs of regression associated with in situ disease may indicate a previously larger invasive primary as a possible source of metastasis.^5^ Review of this patient’s 2023 MIS did indeed show signs of regression and probable isolated melanoma cells in the dermis, but occult invasive melanoma was not detected (Fig 3). The MIS had clear surgical margins and a further wide local excision at the time. The lack of mutations from both the metastatic and 2023 sample also suggests that they may have a common origin. Stage IA melanoma poses a higher risk. A recent review of early-stage melanomas representing as metastatic (50 from a total of 880 melanomas) shows that 11% were initially stage IA. The highest risk of recurrence was within the first 3 years and decreased thereafter.^6^ Theoretically, he could have also had an occult primary metastasizing to the skin. His whole-body Fluorine-18 Fluorodeoxyglucose Positron Emission Tomography/Computed Tomography scan showed multiple metastases but an occult focus could not be cleared labeled. The metastatic disease may have arisen from an occult invasive component of a prior melanoma, a regressed primary, the prior stage IA melanoma, or an occult primary.

The patient had not yet attended cancer genetics at the time of writing. Mutations in CDKN2A, CDK4, BAP1, POT1, or MITF would indicate a hereditary melanoma risk for this patient’s family if discovered.^7^

The case highlights the unexpected recurrence of melanoma as metastatic with an extremely rare paraneoplastic presentation. This diagnosis should prompt a clinician to consider a systemic malignancy screen even when a full skin examination and review of systems is normal.

## Conflict of interest

None disclosed.
